# Evidences of adaptive traits to rocky substrates undermine paradigm of habitat preference of the Mediterranean seagrass *Posidonia oceanica*

**DOI:** 10.1038/srep08804

**Published:** 2015-03-05

**Authors:** Fabio Badalamenti, Adriana Alagna, Silvio Fici

**Affiliations:** 1 CNR-IAMC, Istituto per l'Ambiente Marino Costiero, Via G. da Verrazzano 17, 91014 Castellammare del Golfo (TP), Italy; 2 Dipartimento di Scienze Agrarie e Forestali, Università di Palermo, Via Archirafi 38, 90123 Palermo, Italy

## Abstract

*Posidonia oceanica* meadows are acknowledged as one of the most valuable ecosystems of the Mediterranean Sea. *P. oceanica* has been historically described as a species typically growing on mobile substrates whose development requires precursor communities. Here we document for the first time the extensive presence of sticky hairs covering *P. oceanica* seedling roots. Adhesive root hairs allow the seedlings to firmly anchor to rocky substrates with anchorage strength values up to 5.23 N, regardless of the presence of algal cover and to colonise bare rock without the need for precursor assemblages to facilitate settlement. Adhesive root hairs are a morphological trait common on plants living on rocks in high-energy habitats, such as the riverweed Podostemaceae and the seagrass *Phyllospadix scouleri*. The presence of adhesive root hairs in *P. oceanica* juveniles suggests a preference of this species for hard substrates. Such an adaptation leads to hypothesize a new microsite driven bottleneck in *P. oceanica* seedling survival linked to substrate features. The mechanism described can favour plant establishment on rocky substrates, in contrast with traditional paradigms. This feature may have strongly influenced *P. oceanica* pattern of colonisation through sexual propagules in both the past and present.

Seagrasses are marine flowering plants and represent the most recent stage of a long evolutionary process started from terrestrial monocotyledons. Several morphological and physiological adaptations have allowed terrestrial plants to return to the marine environment in the relatively recent past and to effectively compete with algae^1^,^2^.

Seagrasses belong to the monocotyledon subclass Alismatidae Takht. and are represented by 13 genera that evolved in three separate hydrophilous lineages, i.e., marine Hydrocharitaceae Juss., Zosteraceae Dumort. and the monophyletic group of Posidoniaceae Vines, Ruppiaceae Horan. and Cymodoceaceae Benth. & Hook. f., which offer an extraordinary example of convergent evolution^2^.

Although the morphology and anatomy of seagrasses varies among taxa as a result of different evolutionary pathways, there is a suite of structural adaptations of this group to the marine environment. Among these traits are strap-shaped leaves possessing fibre strands to withstand drag forces exerted by water movements, photosynthesis occurring mainly in the leaf epidermis, loss of stomata and the development of aerenchyma in response to reduced gaseous movements in the liquid medium, submarine pollination and the ability to disperse in the marine environment through several unique mechanisms^3^. Moreover seagrasses need an anchoring system sufficiently developed to withstand the hydrodynamic disturbance produced by waves and currents^1^. This is particularly relevant for species living in high-energy environments.

Although among seagrasses other genera have been reported to grow on hard as well as soft bottoms (i.e *Amphibolis* C. Agardh, *Thalassia* Banks ex K. D. Koenig and *Thalassodendron* Hartog)^3^,^4^, *Phyllospadix* Hook. is the only genus that grows predominantly in the littoral zone on hard substrates where it competes with macroalgae for space. Comparing *Phyllospadix* spp. with the closely related species *Zostera marina* L. - a species that is typically rooted in soft sediment - several anatomical and morphological traits can be identified as adaptive features to rocky substrates and surf exposure^5^. Among these, short and thickened roots with extensive root hairs provide strong anchorage to hard substrates^5^,^6^,^7^. As far as we know *Phyllospadix* *scouleri* Hook is the only species of the genus for which short adhesive root hairs have been described^6^,^7^.

The family Posidoniaceae includes the single genus *Posidonia* K. D. Koenig, which has a disjunct Mediterranean (1 species) and Australian (8 species) distribution^8^. *Posidonia oceanica* (L.) Delile is the dominant seagrass of the Mediterranean Sea, where it is endemic. Its meadows extend from the surface to 40 m depth, with an estimated surface ranging between 2.5 and 5 million hectares^9^. These meadows represent one of the most valuable ecosystems of the Mediterranean Sea and provide essential goods and services to coastal communities^10^.

*Posidonia oceanica* has been described in historical literature as a species growing mainly on soft, nutrient-rich substrates^11^,^12^,^13^. *P. oceanica* meadows are considered the “climax community” of soft sublittoral habitats in the Mediterranean Sea, whose development is facilitated by precursor communities such as *Cymodocea nodosa* Asch. beds that accumulate sediment and organic matter^11^,^12^,^13^. According to these authors, under particular conditions, the development of *P. oceanica* meadows is also possible on rocky reefs but is always preceded by the development of algal turf assemblages that collect sediment and organic matter followed by the development of a *C. nodosa* “pelouse”^11^,^12^,^13^,^14^.

*Posidonia oceanica* propagates via both vegetative and sexual reproduction. Vegetative propagation through rhizome elongation from well-established patches has been widely studied and is considered the dominant process by which seagrasses occupy space and maintain existing meadows^15^,^16^. Recent studies have shown that vegetative fragment recruitment and patch formation does occur^17^ but takes place mainly on complex rocky substrates^18^.

*Posidonia oceanica* flowers during autumn, followed by fruit release in the late spring of the subsequent year^19^. A long-term data series indicates that meadow flowering occurs on average every five years at a basin scale, with massive seed production events recorded every 8–10 years linked to high summer temperatures^19^. Moreover, flowering and fruiting are described as quite regular and diffuse phenomena in the southwestern Mediterranean^20^,^21^. The successful recruitment of *P. oceanica* seeds has been consistently considered a sporadic event in the past^22^. Modern molecular approaches revealed higher genetic variability in meadows than previously thought, leading to the reassessment of the role of sexual reproduction in population dynamics^23^.

One-year survival represents a bottleneck in the seedling life cycle, and successful plantlet recruitment has been recorded mainly on habitats characterised by firm substrates such as rocks covered by algae, dead matte^24^,^25^,^26^,^27^,^28^,^29^ and surprisingly even at very shallow sites (0.5–3 m) characterised by high hydrodynamic regimes^24^,^27^,^28^.

Since 1997, we have observed in several occasions *P. oceanica* seedlings on a variety of substrates at depths ranging from 0.5 to 20 m. The plantlets appeared firmly secured to the sea bottom only when they occurred on consolidated substrates (e.g. rocky reef covered by algae) as they provided strong resistance when pulled^27^,^28^. Seedling found on unconsolidated substrates (e.g. sand or gravel) instead did not offer any resistance when pulled^27^,^28^. Moreover, some seedlings where observed settled even at exposed sites^27^,^28^ and on bare rocks.

Although *P. oceanica* seedling anatomy and ultrastructure have been analysed in detail by several authors^30^,^32^, and the initial seedling development has been observed *ex situ*, in laboratory cultures^31^,^32^, no morphological features have been described thus far that may explain the effective and strong anchorage observed in the field on hard substrates.

Here, the morpho-anatomical and ultrastructural features of *P. oceanica* seedling root systems collected at different locations were analysed to identify specific traits that represent adaptations for establishment on rocky bottoms. Moreover the anchorage strength of seedlings settled on hard substrates in the field was measured. Implications on *P. oceanica* habitat preference and on meadow ecological succession are then discussed.

## Results

### Morpho-anatomical and ultrastructural analyses

**Figure 1:**
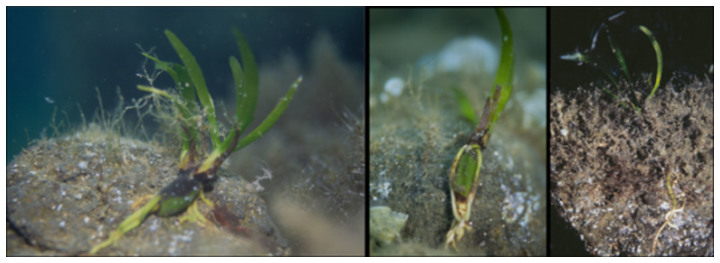
Two- to three-month-old *Posidonia oceanica* seedlings settled on volcanic cobbles at Ustica Island (Tyrrhenian Sea).

No significant differences in morphometric features were detected among seedlings collected at Ustica in July 1997 and at Favignana and Capo Feto in June and July 2004 (P > 0.05). The overall mean values recorded were as follows: seed length 1.93 ± 0.04 (cm ± 1 SE), seed width 0.92 ± 0.02, number of roots 4.50 ± 0.29, maximum root length 2.54 ± 0.21, total number of leaves produced from germination 9.61 ± 0.31, maximum leaf length 5.27 ± 0.21and maximum leaf width 0.53 ± 0.01. The number of standing leaves plus leaf sheaths indicated that seedlings were approximately 2–3 months old (Fig. 1).

**Figure 2:**
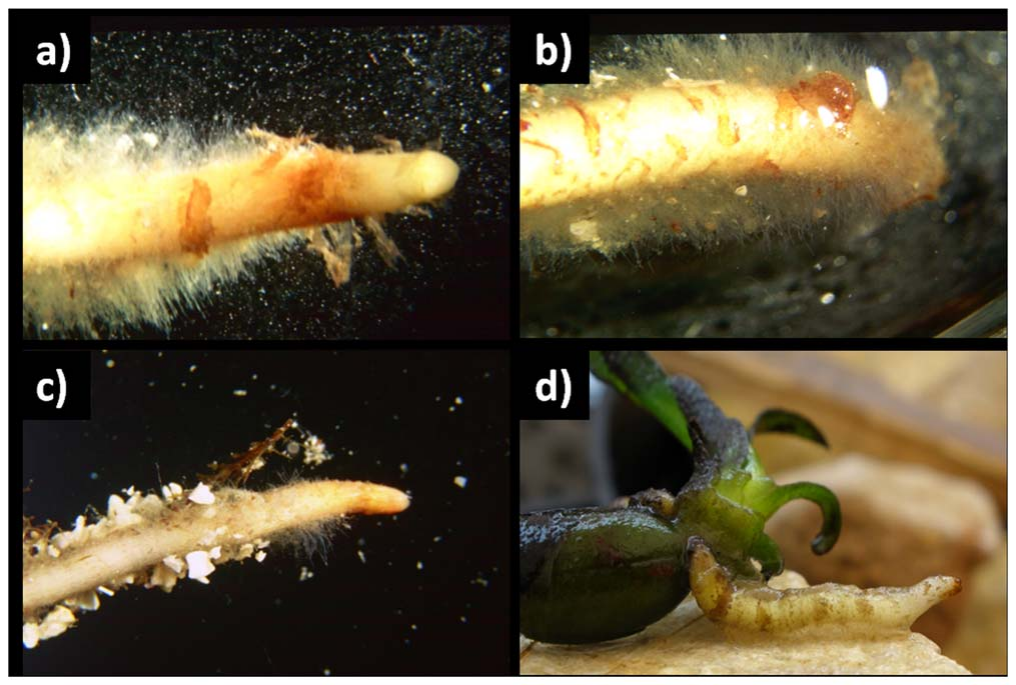
(a), (b) and (c) *Posidonia oceanica* seedling roots observed under the stereomicroscope. (a) and (b) extension of the piliferous zone, (c) sand grains stuck to root hairs, (d) the adhesive coat constituted by the sticky root hairs.

The older portion of the adventitious roots was brown in colour, and the younger portion was cream. Above the root cap, a piliferous zone that extended for several centimetres and covered a large part of the root length was observed (Fig. 2 a, b). Root hairs were covered by a sticky substance that stained positively to PAS, indicating the presence of polysaccharides and glycoproteins. Root hairs constituted an adhesive coat through which roots adhered to the substrate (rocks, algae, and encrusting organisms) (Fig. 1 and 2 d) and to which sand grains remained attached (Fig. 2 c). Adhesive root hairs were recorded on primary and adventitious roots and on the hypocotyl region of the seed. This pattern was consistent across locations.

**Figure 3:**
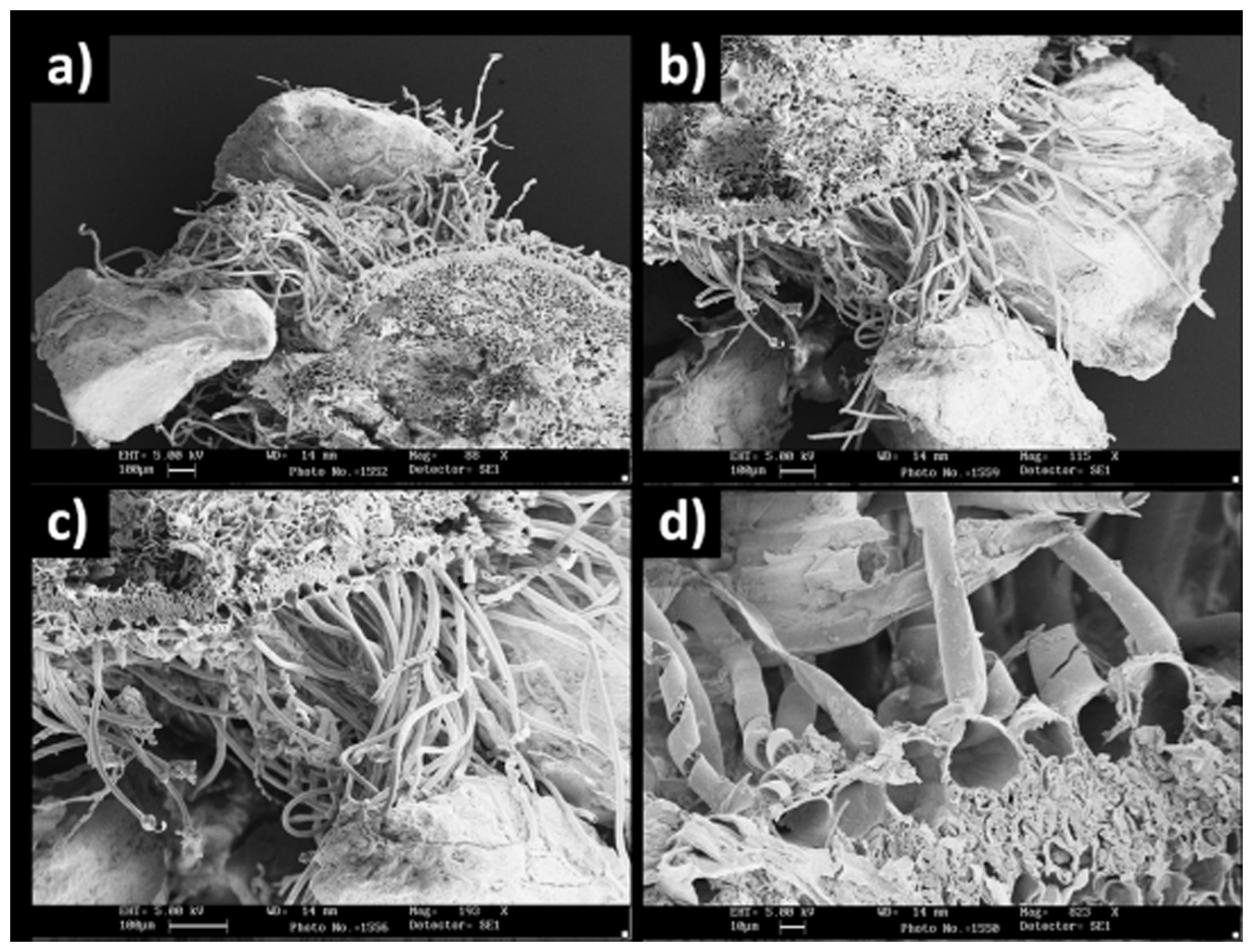
*Posidonia oceanica* seedling root section observed under SEM showing conspicuous root hair coverage originating from epidermal cells, with attached sand grains. (a), (b) details of the root section and of the root hairs stuck to sand grains; (c), (d) close-up of the proximal part of the root hair close to the epidermal cells.

**Figure 4:**
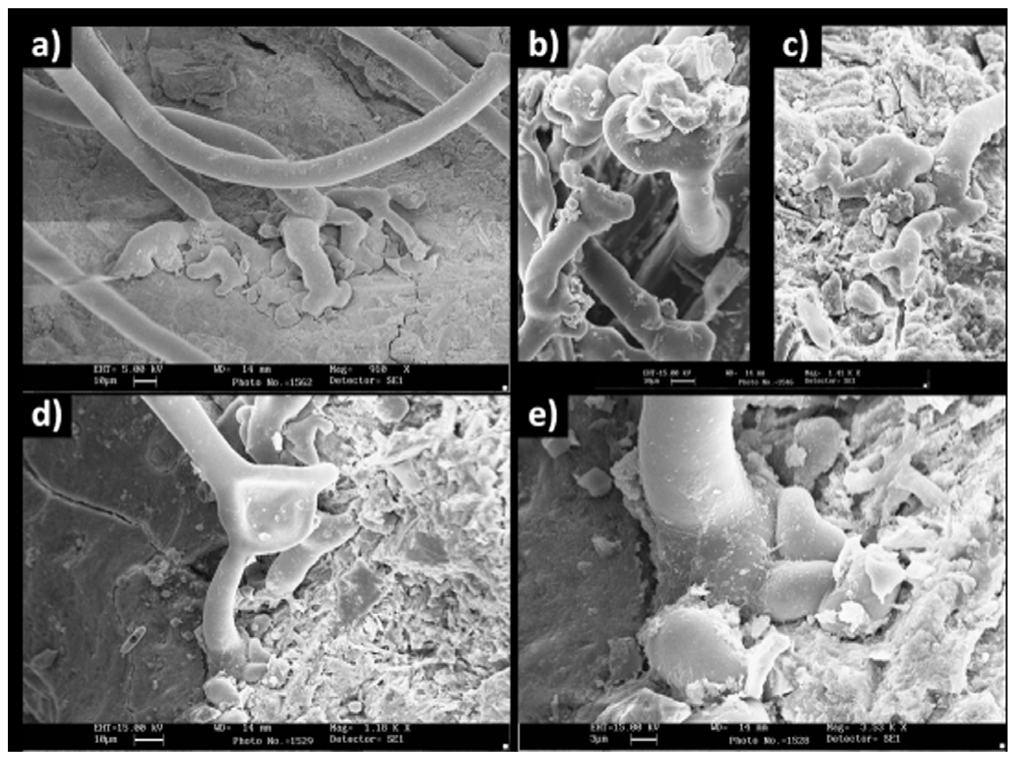
Structural details of *Posidonia oceanica* root hair tips observed under SEM. (a–d) branching of the distal part and lobed root hairs tips; (e) detail of the root hair tip.

Using SEM imaging, the transverse section of adventitious roots from the mature region showed that the epidermis possessed an extensive and dense root hair coverage (Fig. 3). The epidermal cells from which root hairs originated had a mean diameter of 19.46 ± 0.76 (μm±1 SE) [n = 12] (Fig. 3). Root hairs were unicellular and elongated, with a mean length of 707.67 ± 24.09 μm and a maximum length of 2400 μm. The mean root hair width was 12.42 ± 0.36 μm, with a maximum width of 14.17 μm (Fig. 3 and 4). Root hairs were branched or forked distally, with hair tips broadened into an enlarged, lobed, foot-like shape (Fig. 4). This structure had a mean width of 52.70 ± 4.32 μm and a maximum width of 78.33 μm, and it extended the contact area between the hair tip and the substrate (Fig. 4).

### Anchorage strength

The number of standing leaves plus leaf sheaths indicated that seedlings collected at Ustica in 2009 were approximately 5 months old. The force needed to detach seedlings settled on volcanic cobbles ranged from 0.78 to 5.23 N, with a mean value of 2.97 ± 0.42 (N±1 SE). Adhesive root hairs were observed on primary and adventitious roots and on the hypocotyl region of the seeds in all specimens.

## Discussion

This study documents for the first time the presence and morphology of adhesive root hairs in *P. oceanica* seedlings. Adhesive root hairs were recorded in all specimens collected from different substrates along both the mainland and island coasts of northwestern and western Sicily and appeared to be responsible for the strong anchorage strength displayed by seedlings settled on bare cobbles.

Two- to three-month-old seedlings collected at Ustica, Favigana and Capo Feto exhibited similar morphological features and were comparable to specimens of similar age analysed in previous studies^27^,^28^,^30^. Earlier studies did not, however, document the presence of sticky root hairs. Belzunce *et al.*^30^ extensively studied the structure and development of *P. oceanica* seedling root systems of individuals cultured in the laboratory. These authors observed scattered hairs on primary and adventitious roots with a maximum length of 300 μm, less than half the mean length observed in this study, with no sign of adhesive substances.

In our study, seedlings settled on volcanic cobbles were firmly anchored to the substrate, as the force needed to dislodge the plantlets exceeded 2 N on average, which is up to 100-fold higher than that required to uproot seedlings of other seagrasses growing on sand or matte^33^. Seedling root systems conformed to substrate morphology; after detachment, it was possible to observe that roots were adhering directly to the substrate surface rather than via “facilitator” species that provide a settlement surface.

Root hairs have been described for many seagrasses in adult individuals^3^. The density and length of root hairs vary greatly among and within genera, with no clear relationship with features of substrates inhabited by each species^3^. Massive and long root hairs are found in the genera *Thalassia*, *Halophila*, *Zostera* and *Heterozostera*^3^. In *Posidonia*, *Cymodocea*, *Halodule* and *Thalassodendron* root hairs are reported to be rare^34^; however, root hairs have not been recorded in *Amphibolis*^35^. In general, root hairs are transient; the presence of numerous wall ingrowths and plasmodesmata in cells abutting root hairs suggests that they play an important role in nutrient uptake^36^.

Among seagrasses, adhesive root hairs have been reported only for the species *P. scouleri*^6^,^7^. Gibbs^37^ described the emergence of a dense, woolly covering of root hairs near the tip of developing roots in *Phyllospadix* spp. seedlings, similar to that reported in this study for *P. oceanica* seedlings. Adult *P. scouleri* adhesive root hairs are short, thick-walled and lobed and adhere directly to the substrate, allowing the plant to form mat-like colonies in surf-exposed habitats^7^. Histochemical analysis revealed that roots were covered by a PAS-positive, mucilaginous-like substance that is most likely produced by roots and acts as an adhesive^7^.

Among freshwater plants, the presence of adhesive root hairs has been widely described in the riverweed Podostemaceae Rich. ex Kunth^38^,^39^. This family inhabits the extreme habitats of waterfalls and has a highly polymorphic vegetative body. The lower part of the seed, roots and vegetative body present persistent unicellular hairs produced by epidermal cells that secrete a sticky cement once in contact with the substrate^38^. These hairs elongate and become tube-like holdfasts at maturity with an enlarged and branched tip that has a foot-like shape^39^, which is very similar to that documented here for *P. oceanica* seedlings. More recently, the adhesive material was found to be a bacterial biofilm that sticks to the rocky substrate^39^. Podostemaceae “adhesive hairs” are therefore mechanical elements, growing into the upper layer of the biofilm matrix; the broadened hair tips are important for expanding the contact area between the plant and the biofilm^39^.

Typical root hairs in terrestrial and aquatic plants exist for a limited developmental period on a root and function primarily in water and nutrient uptake. The root hairs of *P. oceanica* seedlings described in this study appear quite distinct as they are persistent, at least for some months, and build up an enduring adhesive coat that covers the greater part of the root length, indicating a mechanical, anchoring function. These structures appear more similar to those observed macroscopically in *Phyllospadix* spp. seedlings^37^ and microscopically in species belonging to the Podostemaceae family^39^. The polysaccharidic nature of the adhesive substance can either be associated with plant cell secretions^7^,^40^ or with extracellular polymeric substances (EPS) of bacterial and microalgal biofilms, which are composed of exopolysaccharides^41^. Further investigations are needed to ascertain if *P. oceanica* adhesive root hairs maintain also a nutrient uptake function.

The sticky root hairs in vascular aquatic plants appear to represent an adaptive trait for survival on hard substrates in high-energy environments^39^. The presence of morphologically and functionally similar adhesive root hairs in phylogenetically distant species such as the monocotyledon seagrasses *Posidonia oceanica* (Posidoniaceae) and *Phyllospadix scouleri* (Zosteraceae) and the freshwater dicotyledons Podostemaceae can represent an example of convergent evolution in response to life in habitats exposed to wave action or running water and characterised by hard substrates.

It can be hypothesized that the adhesive properties of *P. oceanica* seedling roots represent an adaptive trait expressed under the right environmental stimuli. In the present work adhesive root hairs were recorded in seedlings collected on different types of substrate i.e. bare cobbles, rock covered by algae and sand, suggesting that substrate type does not play a role in root hair development. This observation was confirmed by a subsequent work (Alagna *et al*., personal observation) in which *P. oceanica* seedlings were reared on soft *vs* hard substrates in an indoor full replicated experimental system. Seedlings produced sticky root hairs in all experimental groups, supporting the hypothesis that substrate type does not influence the development of adhesive root hairs. On the other hand seedling anchorage occurred only on consolidated substrates via root hair adhesion, supporting the hypothesis that sticky root hairs promote early seedling anchorage on consolidated substrates. We cannot exclude that other mechanisms allow *P. oceanica* seedling establishment on other types of substrate, however we failed to observe other mechanisms allowing seedling anchorage on sand.

In aquatic environments vascular plants have evolved specific structures to assist with initial seed and seedling establishment. Among these are the arms covered by stiff bristles of *Phyllospadix* spp. fruits^37^, which help seedlings to get entangled preferentially on intertidal branched algae^42^, and the four-lobed comb of *Amphibolis* spp. seedlings that grapples on adult plants and other structures present on the bottom^43^. Similarly, the presence of an extensive coverage of adhesive hairs on roots allows *P. oceanica* seedlings to adhere to different types of consolidated substrates, such as vegetated and bare rocks; further, adhesive hairs allow seedlings to settle on these substrates even in shallow, exposed sites. Each of these morphological adaptations has been selected and maintained by evolutionary processes because it may increase the probability of a new individual to remain in a favourable habitat for a given species, that has been successfully colonised by adult individuals^44^. In the same way strong anchorage to hard substrates by adhesive root hairs could represent a mechanism of habitat selection in *P. oceanica* seedlings.

Recruitment of juveniles to adult populations is considered a crucial stage in plant life history^45^. Recruitment limitation models identify two main factors potentially affecting population size and species distribution: habitat carrying capacity and the intrinsic ability of a population to grow^46^. These two factors can be differentiated according to the spatial scale of interest. Propagule and suitable microsite availability limits recruitment at the local scale, while dispersal and habitat availability act at the regional scale^46^. Seed limitation and microsite limitation represent the two end-points of a spectrum, with plant populations usually being both seed and microsite limited^45^.

**Figure 5:**
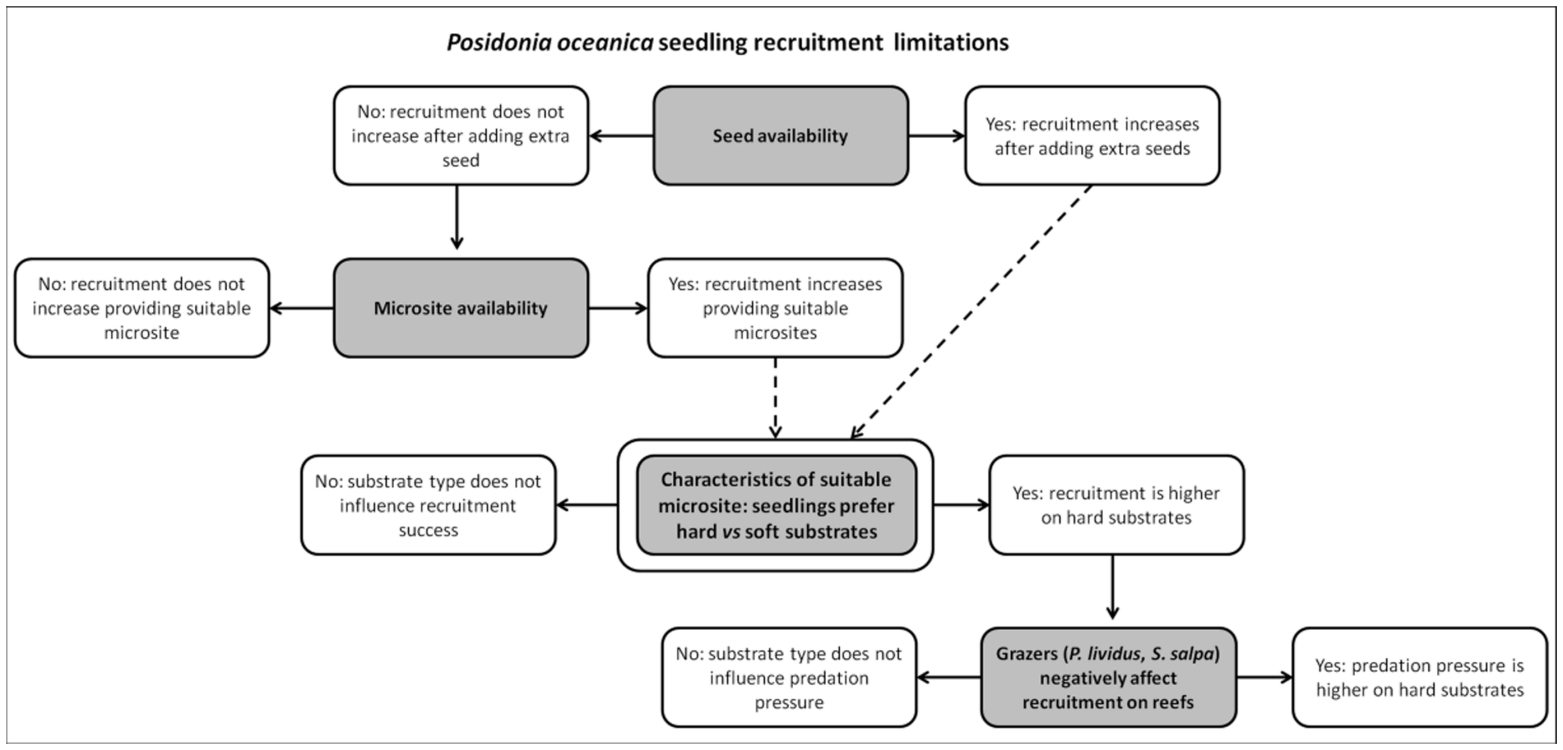
Conceptual diagram showing alternate hypotheses to be tested trough seed sowing experiments in order to (1) evaluate the influence of seed and microsite availability in sexual recruitment limitation of *P. oceanica* populations and to (2) identify characteristics of suitable microsite for *P. oceanica* seedling establishment. Influence of substrate type and predation are highlighted.

*Posidonia oceanica* fruit production is reported to be rare in some region and quite regular in others^20^,^21^, therefore natural levels of seedling supply are expected to vary greatly according to year and location and could represent a bottleneck for recruitment at the local scale (Fig. 5). However, correlative and manipulative experiments have shown that even when seeds are available, recruitment is highly variable (0–70% survival) displaying strong patterning across habitats^25^,^26^,^28^,^29^. These observations point out that microsite availability very likely plays an additional role in limiting *P. oceanica* seedling recruitment (Fig. 5).

Characteristics of suitable microsites for *P. oceanica* seedling recruitment remain to be defined. Substrate type seems to represent a major driver in successful *P. oceanica* seedling establishment, with recruitment mainly occurring on firm substrates such as rock and dead matte with respect to unconsolidated ones such as sand, gravel and pebbles^25^,^26^,^28^,^29^. Plantlets physical dislodgement by hydrodynamic forces is considered one of the main causes for failed seedling establishment^33^,^47^, a suitable microsite is then expected to provide shelter from the drag forces acting on the seedlings and/or to increase the seedling anchoring ability. It can be hypothesized that hard substrates have higher potential compared to soft ones to provide suitable microsite for *P. oceanica* seedling establishment due to the presence of adhesive root hairs. Hence, a new microsite driven bottleneck in *P. oceanica* seedling survival linked to substrate features can be forecasted (Fig. 5). To test this hypothesis a specific manipulative experiment contrasting seedling recruitment success on hard *vs* soft substrates should be run.

Settling on rocky habitats rather than on sand might have negative effects on seedlings survival due to predation by the main consumers of *P. oceanica*, namely the sea urchin *Paracentrotus lividus* (Lamarck, 1816) and the sparid *Sarpa salpa* (Linnaeus, 1758)^48^. It has recently been shown that *P. oceanica* patches growing on rocky matrix are more vulnerable to herbivores than those growing on sandy patches^49^. However, the canopy provided by seedling is smaller if compared to that provided by *P. oceanica* patches or *Cystoseira* spp. forests, therefore sea urchins might not find suitable refuges there, remaining exposed to higher risk of predation^49^. Although *S. salpa* bite marks can be found on *P. oceanica* seedling leaves no evidence of grazing by this herbivore on *P. oceanica* seedlings was found in a predator exclusion experiment^25^. Moreover, sites characterized by the presence of turf usually record a higher sedimentation rate than in *Cystoseira* spp. forest^50^ and represent an obstacle for *P. lividus* movement^51^. It can be hypothesized that seedlings recruiting on turf may thus experience a microsite where sea urchins are less abundant^52^, move slowly^51^ and a desirable amount of sediment is found^50^. Specific manipulative experiments should be run to disentangle the relative importance of the nature of substrate (e.g. vegetated and not vegetated rocky bottoms, sand, etc.) in the presence or absence of herbivores.

The results of the present study have far-reaching ecological implications as they provide new insight into the habitat preferences of *P. oceanica* juveniles, the colonisation potential and distribution of this species and its role in successional series. *P. oceanica*, described in historical literature as a species mainly growing on sandy bottoms^11^,^13^, is shown here to be able to colonise bare rocky substrates without the need for precursor assemblages, in contrast with traditional paradigms. Moreover adhesive root hairs appear to represent an adaptive advantage for early seedling establishment on hard substrates over the sandy ones.

In seagrasses that produce large, fleshy fruits with high dispersal capabilities such as the species belonging to the genus *Posidonia*, sexual propagation represents the colonisation strategy that acts over larger spatial scales (hundreds of kilometres, according to Kendrick *et al*.^53^), and seedlings are expected to be responsible for the initial establishment of the meadow^54^. The strong anchorage displayed by *P. oceanica* seedlings on hard substrates provided by adhesive root hairs leads us to predict a more successful recruitment by sexual propagules on hard bottoms respect to the soft ones, in accordance with the findings of several studies^24^,^26^,^27^,^28^. If this hypothesis would be confirmed, we could reasonably expect that the patterns of colonisation by sexual propagules of this species have been strongly influenced by the availability and distribution of hard substrates at the regional and basin scale. This, in turn, could shed light on current and historical patterns of recolonisation, such as after the last Pleistocene ice age^55^, when populations persisting in relict zones began to spread to suitable areas after the subsequent sea level rise.

## Methods

### Study area

Observations were made on *P. oceanica* seedlings found along the northwestern and western coasts of Sicily (Italy) at Favignana Island (37°55′59″N; 12°19′13″E), Ustica Island (38°43′0″N; 13°11′18″E) and Capo Feto (37°39′20″N, 12°32′7″E). Seedlings were collected in July 1997 at Ustica and in June and July 2004 at Favignana and Capo Feto in shallow waters (1–3 m). At Ustica seedlings were anchored on large cobbles, on *Cystoseira* spp. stands or laid down on sand. At Favignana and Capo Feto seedlings were anchored on bedrocks covered by macroalgal turf and *Cystoseira* spp. stands or laid down on sand. With the exception of those collected on sand - that were not anchored- all seedling offered a resistance when collected as they were attached to the substrate. We conducted a specific measurement of the seedling anchorage strength in October 2009 at Ustica, on specimens settled at a depth of approximately 3 m on volcanic cobbles, no seedlings were recorded on other substrates in this occasion.

### Morpho-anatomical and ultrastructural analyses

Morpho-anatomical and ultrastructural analyses were performed on seedlings collected between June and July at Ustica (1997), Favigana (2004) and Capo Feto (2004), where possible without separating the seedlings from the substrate. Seedlings were transported to the laboratory, photographed using a stereomicroscope, and fixed in a 5% buffered formalin/seawater solution. Seed size, number of roots, maximum root length, total number of leaves produced from germination, maximum leaf length and width were recorded (n = 12 at each location). Variation among locations was tested for each variable using one-way ANOVA.

Subapical sections of adventitious roots were obtained from specimens collected at Ustica (1997) on bare cobbles and at Capo Feto on rocks covered by algae and on sand (2004). Root sections were dehydrated in a tertiary butyl alcohol series and processed in paraffin. Next, the samples were mounted on SEM stubs, sputter-coated with gold, and examined using a Leica 5420 SEM at a 15 KV operating voltage to analyse morpho-anatomical features of seedling roots and root hairs. Root-hair length and width and hair-tip morphology and width were recorded (n = 12).

Structures observed under the stereomicroscope and SEM were compared with those reported in the literature for seedlings of *P. oceanica* and for other aquatic phanerogams.

To obtain a first chemical characterization of the adhesive substance covering root hairs, the whole mounts of seedling roots were stained with Periodic acid-Schiff (PAS) to search for the presence of polysaccharides and glycoproteins.

### Measurement of anchorage strength

The anchorage strength was measured on seedlings settled on bare volcanic cobbles at Ustica Island in October 2009. Twelve cobbles (greatest diameter: mean 16.50 ± 0.60 SE cm), each with an attached seedling, were haphazardly chosen and carefully moved from the seafloor into 50 l plastic boxes on board the research vessel. The force needed to detach seedlings from the substrate was immediately measured and used as a proxy for anchorage strength. A hook-shaped plastic-coated iron wire 3 mm in diameter was carefully passed underneath the seed body. One end of the wire was connected to a digital spring scale (Vetek, precision 5 g). Measurements (n = 12) were taken by pulling the seedling perpendicular to the substrate until detachment while an operator kept the cobble still at the bottom of the box. The pull weight was converted to force (Newtons). Seedlings were then transported to the laboratory and stored frozen for morphological analysis (seed size, number of roots, maximum root length, total root length, total number of leaves produced from germination, maximum leaf length and width).

## Author Contributions

All authors contributed equally to this work. F.B. designed the study and collected data, F.B. and S.F. wrote the first draft of the introduction, A.A. wrote the main paper. All authors discussed the results and implications and commented on the manuscript at all stages.
